# Spinal cord hypometabolism associated with infection by human T-cell lymphotropic virus type 1(HTLV-1)

**DOI:** 10.1371/journal.pntd.0006720

**Published:** 2018-08-27

**Authors:** Luiz C. F. Romanelli, Débora M. Miranda, Anna B. F. Carneiro-Proietti, Marcelo Mamede, Herika M. M. Vasconcelos, Marina L Martins, Anísia S. D. Ferreira, Daniela V. F. Rosa, Jonas J. Paula, Marco A. Romano-Silva, Rodrigo Nicolato

**Affiliations:** 1 Molecular Medicine Postgraduate Program, Universidade Federal de Minas Gerais (UFMG), Belo Horizonte, Minas Gerais, Brazil; 2 HTLV-1 Research Interdisciplinary Group (GIPH), Hemominas Foundation, Belo Horizonte, Minas Gerais, Brazil; 3 Departament of Pediatrics, Universidade Federal de Minas Gerais (UFMG), Belo Horizonte, Minas Gerais, Brazil; 4 Departament of Mental Health, Universidade Federal de Minas Gerais (UFMG), Belo Horizonte, Minas Gerais, Brazil; Universidad Nacional Mayor de San Marcos, PERU

## Abstract

**Background:**

HTLV-1 infection is endemic in Brazil. About 1 to 2% of the Brazilian population is estimated to be infected, but most infected HTLV-1 individuals do not know about their own infection, which favors the continuity of sexual and vertical virus transmission. In addition, HTLV-1 associated central nervous system diseases and their pathophysiologic mechanisms are not fully understood. This study aimed to evaluate the correlation of spinal cord metabolism, viral and inflammatory profiles with features of neurological presentation in HTLV-1 infected individuals.

**Methodology:**

This is a cross-sectional study of a cohort including 48 HTLV-1 infected individuals clinically classified as asymptomatic-**AG** (N = 21), symptomatic-**SG** (N = 11) and HAM/TSP-**HG** (N = 16) and a nested case-control study with HTLV-1 infected individuals-**HIG** (N = 48) and HTLV-1 non infected controls-**CG** (N = 30) that had their spinal cord analysed by Positron Emission Tomography with 18F-Fluordeoxyglucose (18F-FDG PET/CT). HTLV-1 infected individuals had 18F-FDG PET/CT results analyzed with clinical and demographic data, proviral load, cytokines and chemokines in the blood and cerebrospinal fluid (CSF).

**Principal Findings:**

18F-FDG PET/CT showed hypometabolism in the thoracic spinal cord in HTLV-1 infected individuals. The method had an accuracy of 94.4% to identify HAM/TSP. A greater involvement of the thoracic spinal cord was observed, although hypometabolism was also observed in the cervical spinal cord segment in HTLV-1 infected individuals. Individuals with HAM/TSP showed a pro-inflammatory profile in comparison to asymptomatic and symptomatic groups, with a higher level of Interferon-inducible T-cell alpha chemoattractant (ITAC/CXCL_11_), IL-6, IL-12p70 in the plasma; and ITAC, IL-4, IL-5, IL-8 (CXCL_8_) and TNF-alpha in the CSF. Using regression, thoracic spinal cord SUV (standardized uptake value) and CSF ITAC level were identified as the HAM/TSP predictors in the multivariate model.

**Conclusions:**

18F-FDG PET/CT imaging showed spinal cord hypometabolism in most HTLV-1 infected individuals, even in the asymptomatic HTLV-1 group. Thoracic spinal cord hypometabolism and CSF-ITAC levels were identified predictors of HAM/TSP.

**Significance:**

Our findings suggested that in most HTLV-1 infected individuals there was compromise of central nervous system (CNS) structures despite of the lack of clinical symptoms. To explain the found hypometabolism, the role of microcirculatory and metabolic factors in the pathogenesis of neurological diseases associated with HTLV-1 infection must be further investigated. It is paramount to evaluate the central nervous function and to compare the performance among HTLV-1 infected individuals considered asymptomatic to the uninfected controls.

## Author summary

For the past 30 years, human T-cell lymphotropic virus type-1 (HTLV-1) has been isolated and associated with neoplastic, inflammatory, and infectious diseases. It is known that the neurological disorder associated with HTLV-1 comprises HTLV-1-associated myelopathy (HAM/TSP) or any other isolated signals and symptoms. Despite all the knowledge accumulated so far, the association of neurological diseases to HTLV-1 infection remains difficult and neglected. We designed this study in order to assess the degree of neurological impairment associated with HTLV-1 infection through a metabolic evaluation with 18F-FDG PET/CT. Our results evidenced a more pronounced hypometabolism in the spinal cord of individuals with neurological impairment, but also evidenced hypometabolism in asymptomatic HTLV-1 infected individuals. We believe that areas of the CNS with lower circulatory and perfusional balance are more vulnerable to HTLV-1 infection. Mechanisms of cellular entry of the virus may be associated with loss of microcirculatory homeostasis and predisposition to a breakdown of the blood-brain barrier in these areas. Further studies are still necessary to shed light on the mechanisms associated with brain and spinal hypometabolism.

## Introduction

The Human T-cell lymphotropic virus (HTLV-1) was associated with tropical spastic paraparesis (TSP) and HTLV-1 associated myelopathy (HAM) in the eighties [1–2]. Both diseases were recognized as the same neurological disorder in 1988 and called HAM/TSP [3]. Since then, a wide range of inflammatory, neoplastic, and infectious diseases have been associated with HTLV-1 infection [4]. Different neurological impairments have been described in HTLV-1 infection without meeting the criteria defined for HAM/TSP, although enough to compromise labor capacity and social life of infected individuals [5–6].

The combination of specific virus characteristics and the varied elicited individual immune response by the host leads to HAM/TSP in only 3% of infected individuals [7]. Female gender, transmission pathway, high proviral load and proinflammatory immune response are risk factors for the development of neurological disease [8]. The pathophysiological mechanisms of central and peripheral nervous system impairment caused by HTLV-1 are not fully understood. Many years may elapse from the first clinical symptoms to a clear diagnosis of HAM/TSP. There is a total absence of accurate markers to define the established neurological disease associated with HTLV-1. In order to have a clear diagnosis of HTLV-1-associated myelopathy (HAM/TSP), it is necessary a long follow-up and exclusion of other causes of myelopathy [9–10].

The most common and non-specific finding observed in thoracic spinal cord magnetic resonance imaging (MRI) is atrophy of the thoracic spinal cord, which appear several years after disease onset [11]. In a study conducted in our cohort (HTLV Research Interdisciplinary Group—GIPH), visual inspection of spinal cord MRI had low sensitivity for diagnosis of HAM/TSP in early and intermediate phases of the disease [12]. Some authors have reported that magnetic resonance imaging was more sensitive to evidence the presence of atrophy and focal lesions in the spinal cord, which also demonstrated a relationship between the duration of the disease and the degree of spinal cord atrophy [13–15].

HTLV-1 infection is chronic over decades, and may evolve with milder neurological impairment of defined HAM/TSP. The differential diagnosis of HTLV-1 milder neurological impairment is often neglected or confused. In order to evaluate central nervous system involvement, we designed this study to analyze blood and cerebrospinal fluid immunomarkers and thoracic spinal cord 18F-FDG PET/CT. Neuronal activity and function are measured indirectly through metabolism of glucose by 18F-FDG PET/CT [16]. PET has been used for the differential diagnosis of primary, opportunistic and neoplastic CNS lesions in human immunodeficiency virus (HIV) carriers. In HIV-infected individuals with cognitive impairment the 18F-FDG PET has shown subcortical hypermetabolism in the basal ganglia, striatum and thalamus, with discretely increased metabolism in studies following the introduction of combined antiretroviral therapy [17]. We hypothesized that in the early stages of HTLV-1 spinal cord impairment, 18F-FDG PET/CT would show a metabolic increase due to inflammation. On the other hand, in advanced phases of HAM/TSP, a degenerative phase with neuronal loss, a lower metabolism would be present and the asymptomatic individuals would have a metabolism close to that of uninfected controls. The data was analyzed using a regression model to determine predictive markers of the HAM/TSP.

## Results

### Demographic and clinical data

Subjects of this study were grouped according to their clinical neurological conditions. Significant statistical difference was not observed between groups with respect to age and follow-up time at GIPH. There was a significant difference regarding gender due to the higher proportion of female in the symptomatic and HAM/TSP group. The degree of neurological impairment was measured using the Expanded Disability Status Scale (EDSS) [18], which showed a significant difference between infected HTLV-1 groups and a strong correlation with the Ambulation Index [19]. Demographic data are summarized in Table 1.

**Table 1:** Demographic data of HTLV-1 infected patients and control group.

		**GENDER**	**AGE**	**EDSS SCORE**	**GIPH TIME**
**Groups**	**N (%)**	**Female (%)**	**Years** **Mean SD**	**Interval (M_e_)**	**Years** **Mean SD**
**AG**	21 (27)	9 (43)	51 (10)	0–0 (0)	11 (5)
**SG**	11 (14)	9 (82)	46 (13)	0–2 (1)	10 (6)
**HG**	16 (21)	13 (81)	51 (17)	2–8 (5)	9 (5)
**CG**	30 (38)	17 (57)	47 (15)	NA NA	NA NA
**Total**	78 (100)	48 (62)	49 (14)	0–8 (1)	10 (5)
	***P***	0.045^a^	0.689^b^	< 0.001^c^	0.186^c^
		**GENDER**	**AGE**	**EDSS SCORE**	**GIPH TIME**
**Groups**	**N (%)**	**Female (%)**	**Years** **Mean SD**	**Interval (M_e_)**	**Years** **Mean SD**
**HIG**	48 (62)	31 (67)	51 (11)	0–8 (1)	10 (5)
**CG**	30 (38)	17 (57)	47 (15)	NA NA	NA NA
**Total**	78 (100)	48 (62)	49 (14)	0–8 (1)	10 (5)
	***P***	0.633^a^	0.486^d^	NA	NA

EDSS—Expanded disability status scale; SD—Standard deviation; NA—Not applicable

^a^ Chi-square test—Exact of Fisher;

^b^ ANOVA;

^c^ Kruskal Wallis test;

^d^ t-test.

AG = Asymptomatic Group, SG = Symptomatic Group, HG = HAM/TSP, CG = Control Group, HIG = HTLV-1 infected Group

**Fig 1:**
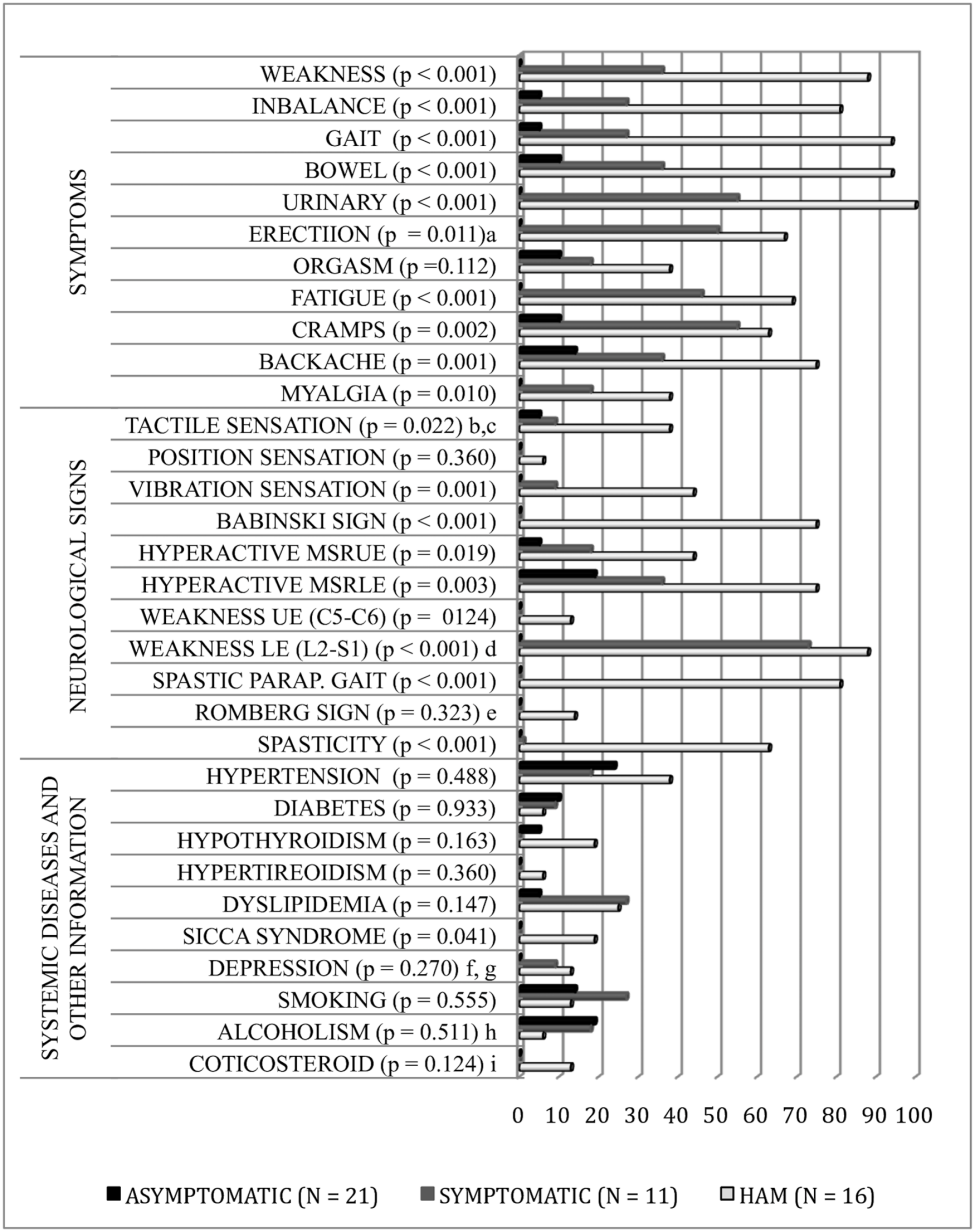
Percentages of symptoms and neurological signs, systemic diseases and social habits by group of HTLV-1 infected. MSRUE—Muscle stretch reflexes of the upper extremities; MSRLE—Muscle stretch reflexes of the lower extremities; MSR—Muscle stretch reflexes; UE—upper extremities; LE—lower extremities; ^a^Considered men only; ^b^Sensory changes due to leprosy sequel (AG); ^c^Hypoaesthesia below T1 with normal MRI (SG). It evolved with spontaneous clinical improvement; ^d^Muscle weakness compromised the myotomes in the following proportion: L2 (88%), L4-L5 (38%), S1 (25%) and L3 (13%) in HG and L2 (73%) in SG; ^e^It was not evaluated in 2 restricted individuals the wheelchair (HG); ^f^The prevalence of depression may be underestimated. Participants of the GIPH cohort with uncontrolled psychiatric illness and high doses of psychotropic medications were not invited for this study. ^g^Geriatric Depression Scale– 15 itens (GDS -15), ^h^WHO (>15 doses/week-men and >10 doses/week-women), dose—8 a 13 g of ethanol; ^I^0.25 e 0.75 mg dexamethasone/day. AG = Asymptomatic Group, SG = Symptomatic Group, HG = HAM/TSP, CG = Control Group.

Neurological symptoms and signs were statistically different between HTLV-1 infected groups. Prevalent concomitant clinical diseases, smoking and alcoholism were not statistically different between HTLV-1 infected groups, except sicca syndrome, with 19% prevalence in the HAM/TSP group. Only two HAM/TSP patients were in use of low-dose corticosteroids. The prevalence of depression was investigated in HTLV-1 infected individuals using the Geriatric Depression Scale– 15 items (GDS -15) [20] and may be underestimated, since individuals on psychotropic drug treatment were not invited to participate in this study. Fig 1 shows the percentage of symptoms and neurological signs, systemic diseases and social habits in each of the HTLV-1 infected groups. In the studied group, HAM/TSP patients evolved with slow progression. The disease duration in years ranged from one to 35 years, with a mean of 12 years (SD 10) in the HAM/TSP group and ranged from 6 months to 9 years, with a mean of 3 years (SD 3) in the symptomatic group.

### Spinal cord standardized uptake value (SUV)—18-F FDG PET/CT

A reduction in metabolism in the spinal cord was observed in the 18-F FDG PET/CT images in HTLV-1 infected groups compared to uninfected controls and the degree of hypometabolism correlated with the degree of neurological impairment. Thoracic spinal cord SUV and EDSS score had a negative correlation r_s_ = —.526, R^2^ = 0.28, p < .001.

**Fig 2:**
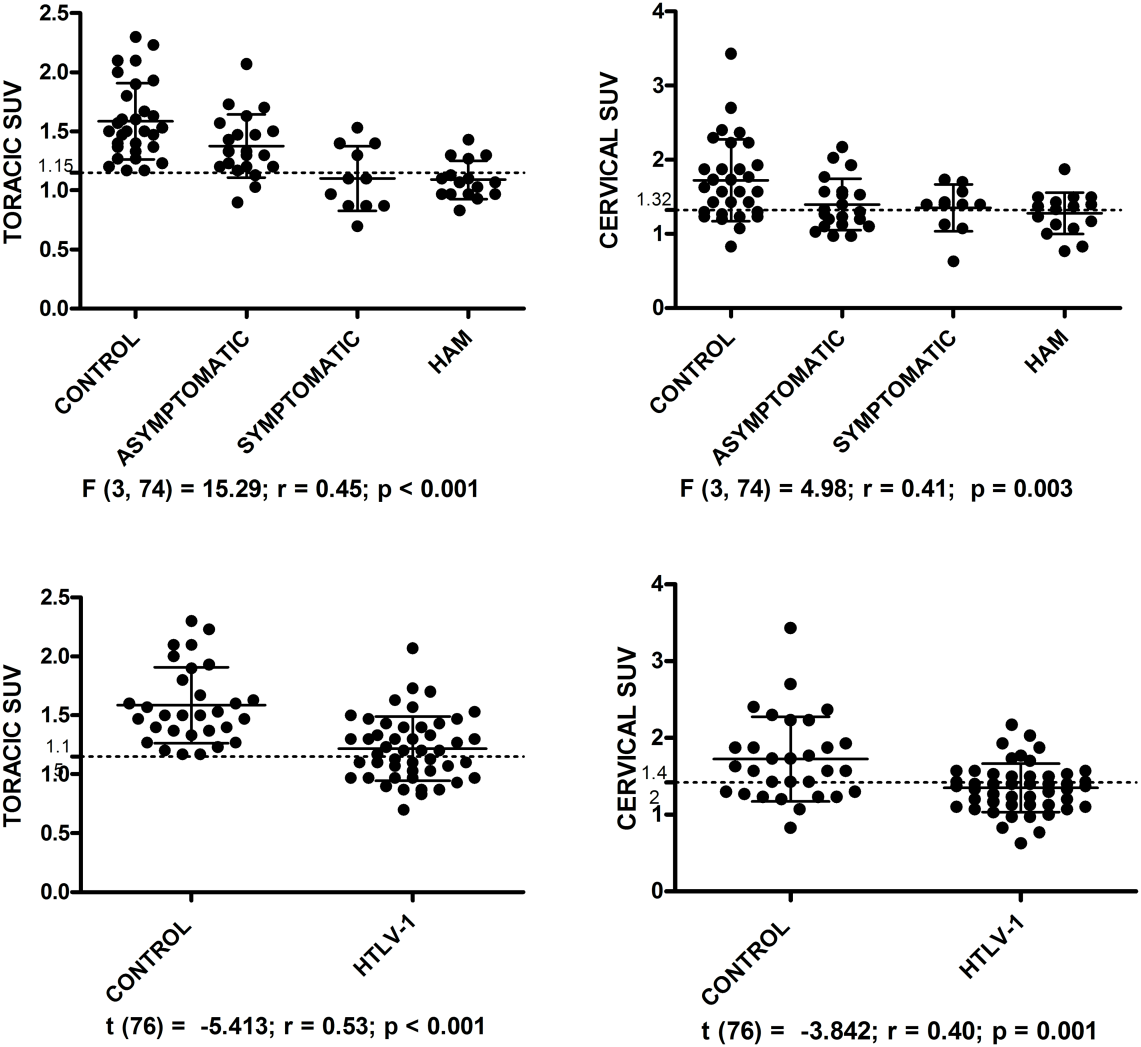
Graphs of standardized uptake value (SUV) in thoracic and cervical spinal cord of HTLV-1 infected individuals and control group. The dotted lines represent the cutoff points to discriminating between the HAM/TSP and asymptomatic and HTLV-1 infected and control groups.

Significant statistical difference was not observed between gender with respect to cervical (p = 0.514) and thoracic (p = 0.602) spinal cord SUV. Thoracic and cervical SUV were significant difference between the four groups, and between the HTLV-1 infected and the control group (Fig 2).

In the multiple comparison between groups, post ROC analysis, there was significant difference between the control group compared to asymptomatic, p = 0.041 (CI 95%, 0.01–0.65) and HAM/TSP, p = 0.006 (CI 95%, 0.10–0.78) in the cervical spinal cord segment and between the control group and all HTLV-1 infected groups: asymptomatic group, p = 0.043 (CI 95% 0.005–0.415); symptomatic, p < 0.001 (95% CI 0.232–0.740); and HAM/TSP, p < 0.001 (CI 95% 0.273–0.719) in the thoracic spinal cord segment. The asymptomatic and HAM/TSP groups also showed a significant difference in the thoracic spinal cord SUV, p = 0.012 (CI 95% 0.047–0.525). Thoracic spinal cord SUV of the symptomatic group was similar to that observed in HAM/TSP group, p = 1.00. Thoracic spinal cord SUV presented accuracy of 94.4%, 85.7% and 81.6%, in the correct classification of groups: HAM/TSP and control (p < 0.001; CI 95% 0.89–1.00), HAM/TSP and asymptomatic (p = 0.001; CI 95% 0.72–0.97) and HTLV-1 infected and control (p < 0.001 CI 95% 0.72–0.91), respectively. In the ROC curve, thoracic spinal cord SUV ≤ 1.15 discriminated individuals of HAM/TSP group with 75% sensitivity and 100% specificity in relation to the control group and with 76.5% sensitivity and 85.7% specificity in relation to the asymptomatic group and cervical spinal cord SUV ≤ 1.32 discriminated HAM individuals with 68.8% sensitivity and 80% specificity in relation a control group. Cervical spinal cord SUV ≤ 1.42 discriminated between HTLV-1 infected and control groups, with accuracy of 72.2%, 64,6% sensitivity, 70% specificity, p = 0.001; CI 95% 0.60–0.84. Cervical spinal cord SUV was not able to discriminate between the HTLV-1 infected groups. Another point observed in the cervical and thoracic SUV was a progressive reduction in the standard deviation as the neuronal impairment increased, higher in control group and lower in HAM/TSP group.

### Blood and CSF proviral load

**Fig 3:**
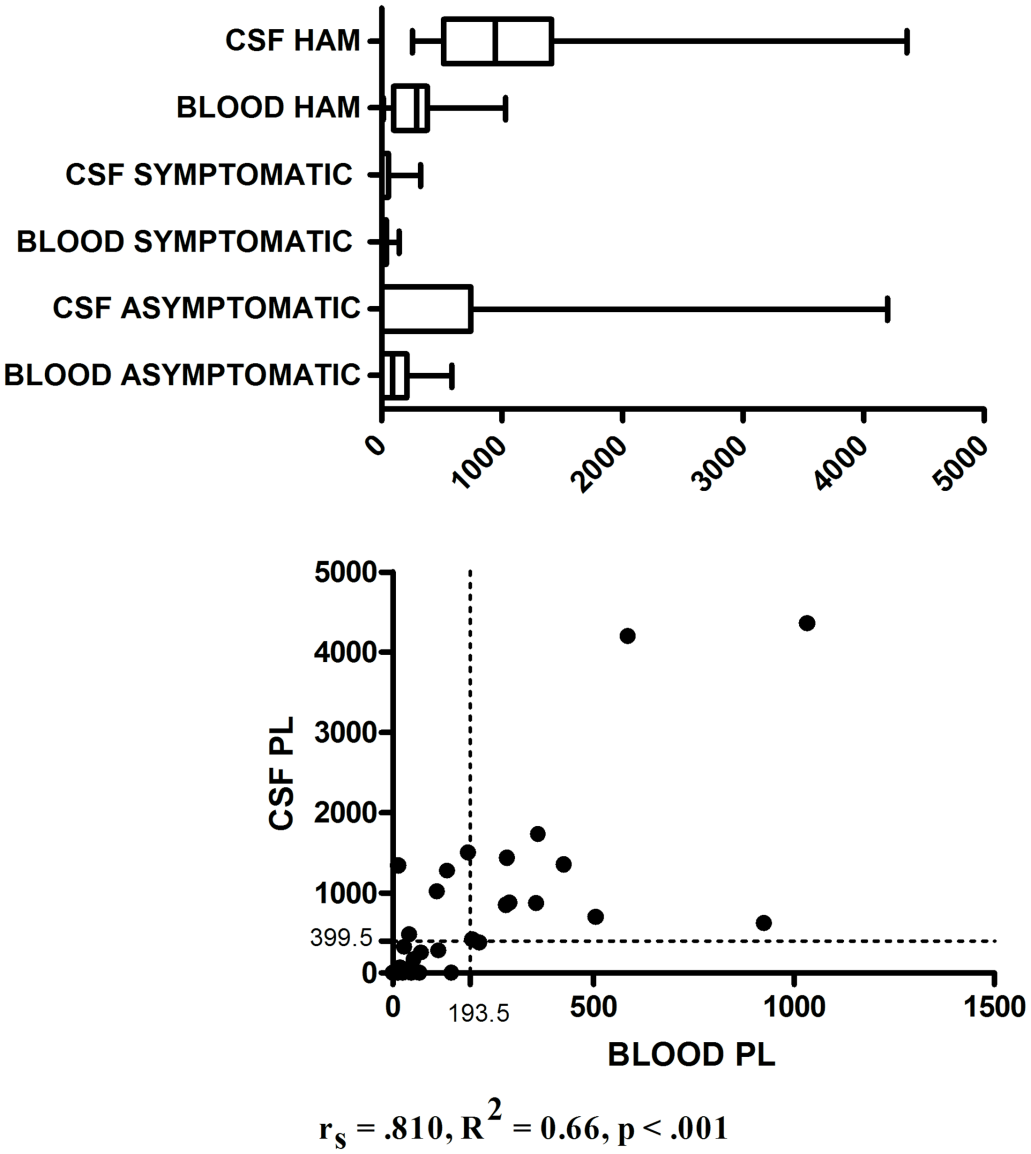
Blood and CSF proviral load (copies/10^3^ cells): distribution by group (above) and correlation (below). The dotted lines represent the proviral load cutoff points to discriminating between HAM/TSP and asymptomatic groups.

Blood (p = 0.566) and CSF (p = 0.721) proviral load distribution did not show a gender difference. There was a statistically significant difference in the mean proviral load of blood and CSF among the three HTLV-1 infected groups: 142 (SD 173; M_e_ 90; N 20) asymptomatic; 29 (SD 42; M_e_ 13; N 11) symptomatic; 336 (SD 301; M_e_ 292; N 14) HAM/TSP in the blood and 515 (SD 1035; M_e_ 0; N 18) asymptomatic; 50 (SD 105; M_e_ 0; N 11) symptomatic; 1228 (SD 1086; M_e_ 946; N12) HAM/TSP in the CSF, p < 0.001, copies/10^3^ cells (Fig 3). Positive correlation between blood and CSF proviral load was observed, r_s_ = .810, R^2^ = 0.66, p < .001 (Fig 3). Proviral copies in CSF were not detected in 10/21 asymptomatic (55.6%) and 7/11 symptomatic (72.2%), although were detected in all HAM/TSP individuals. In these individuals with proviral load not detectable in the CSF, the proviral load in blood samples was also low: 15.7 ± 24.3 (0–67) copies/10^3^ cells in the asymptomatic; 25.2 ± 49.6 (0–146) copies/10^3^ cells in the symptomatic. On the other hand, CSF proviral load was higher than blood proviral load in all individuals with detectable CSF proviral load, except in an individual of the HAM/TSP group in which CSF proviral load was 33% lower than blood. In the other subjects with proviral load detectable in the CSF (N = 22), the ratio between CSF and blood proviral load showed an average increase of 9 ± 19.6 (1.38 to 95.64) times, median of 3.5 times. There was no statistically significant difference between the groups with regard to CSF and blood proviral load ratio (p = 0.670): N = 8, 4.6 ± 3.1 (1.4–9.4) times, median of 3.1 times in the asymptomatic group; N = 3, 5.3 ± 5.2 (1.4–11.2) times, median of 3.2 times in the symptomatic; and N = 11, 13.2 ± 27.5 (2.1–95.6) times, median of 4.2 times in the HAM/TSP group. Blood proviral load **≥** 193.5 and CSF proviral load **≥** 399.5 discriminated individuals between HAM/TSP and asymptomatic groups, with accuracy of 74,1%, 71,4% sensitivity, 75% specificity, p = 0.018; CI 95% 0.573–0.909 and accuracy of 81.5%, 91.7% sensitivity, 72.2% specificity, p = 0.004; CI 95% 0.660–0.969, respectively.

### Cytokine and chemokine biomarker

The distribution of cytokines in relation to gender did not present significant statistical difference in HAM/TSP group. Plasmatic Fracktal, IFN-gamma, IL-12p70, IL-13, IL-17 A, IL-1 beta, IL-2, IL-21, IL-6, IL-7, TNF-alpha, CSF Fracktal and IL-7 presented a statistically significant difference (p < 0.05) in relation to gender in asymptomatic group and plasmatic IL-17A, IL-21 and CSF Fracktal in the symptomatic group. The cytokines that presented significant statistical difference between HTLV-1 infected groups were: ITAC (r = 0.43), IL-12p/70 (r = 0.26), IL- 17 A (r = 0.28) in the plasma, and ITAC (r = 0.41), IFN-gamma (r = 0.25), IL-5 (r = 0.33), IL-8 (r = 0.34) and TNF-alpha (r = 0.32) in the CSF (Table 2).

**Table 2:** Blood and CSF cytokines of HTLV-1 infected individuals.

GROUPS	AG (N = 21)		SG (N = 11)		HG (N = 15)		
Plasma (pg/ml)	Mean (SD)	M_e_	Mean (SD)	M_e_	Mean (SD)	M_e_	p^a^
**ITAC**	11.3(8.8)	8.8	10.0(5.0)	8.3	16.3(8.6)	15.2	0.003
**GMCSF**	28(63)	6.5	19.1(16.2)	13.5	14.1(12.7)	9.4	0.395
**Fracktalkine**	66(36)	68.5	57.1(26.2)	52.7	53.0(21.1)	50.1	0.241
**INF-gamma**	7.7(11.7)	5.3	5.6(2.7)	5.8	4.6(1.9)	4.2	0.193
**IL-10**	1.1(0.5)	0.9	1.1(0.6)	0.9	1.0(0.6)	0.7	0.652
**Mip3alpha**	4.5(1.9)	3.7	4.5(2.2)	3.8	3.7(0.5)	3.8	0.289
**IL-12p70**	1.2(1.1)	0.7	0.8(0.8)	0.4	1.7(1.1)	1.9	0.035
**IL-13**	2.1(1.8)	1.7	1.9(1.2)	1.7	1.7(1.5)	1.3	0.665
**IL-17-A**	5.5(3.3)	4.7	5.0(3.0)	4.5	3.5(1.6)	3.1	0.027
**IL-1beta**	0.6(0.4)	4.9	0.6(0.2)	0.6	0.6(0.1)	0.6	0.079
**IL-2**	1.9(1.8)	1.1	1.7(1.0)	1.2	2.0(1.1)	1.5	0.172
**IL-21**	2.0(4.9)	0.7	0.8(0.5)	0.9	0.8(0.4)	0.7	0.987
**IL-4**	3.1(3.1)	1.7	2.3(1.7)	2.2	1.8(1.1)	1.4	0.360
**IL-23**	71(41)	67.7	67.1(29)	58.2	62.8(19.1)	60.8	0.552
**IL-5**	0.6(0.5)	0.4	0.7(0.3)	0.5	0.5(0.2)	0.4	0.724
**IL-6**	0.7(0.4)	0.7	0.7(0.5)	0.6	1.0(0.6)	1.0	0.065
**IL-7**	1.0(1.3)	0.3	0.4(0.4)	0.2	0.7(0.6)	0.3	0.630
**IL-8**	2.3(1.4)	2.0	2.1(1.5)	1.5	2.0(0.9)	2.0	0.343
**TNF-alpha**	0.9(0.8)	0.6	0.5(0.2)	0.5	0.8(0.6)	0.7	0.797
**CSF (pg/ml)**	**AG (N = 21)**		**SG (N = 11)**		**HG (N = 14)**		
**ITAC**	58.3(32.3)	51.7	55(32.3)	44.4	117(64.2)	100.5	0.002
**GMCSF**	0.7(0.2)	0.7	0.8(0.2)	0.8	0.8(0.3)	0.7	0.317
**Fracktalkine**	141(32.8)	131.5	155(26.1)	148.1	134(32.2)	142.7	0.489
**INF-gamma**	1.0(0.9)	0.6	1.8(1.7)	0.6	3.0(3.3)	2.0	0.043
**IL-10**	4.3(2.8)	4.4	4.8(5.8)	2.7	5.3(2.4)	5.4	0.250
**Mip3alpha**	33.0(6.5)	31.8	30.9(5.7)	32.8	30.8(7.7)	28.5	0.207
**IL-12p70**	1.1(0.9)	0.8	1.0(0.5)	0.8	1.6(1.0)	1.2	0.108
**IL-13**	4.3(2.5)	5.0	5.4(2.2)	5.7	4.2(2.7)	5.1	0.879
**IL-17-A**	0.3(0.4)	0.3	0.2(0.1)	0.2	0.2(0.1)	0.2	0.574
**IL-1beta**	0.3(0.4)	0.2	0.3(0.5)	0.3	0.3(0.02)	0.3	0.574
**IL-2**	1.1(0.3)	1.2	1.1(0.7)	1.2	1.3(0.4)	1.2	0.288
**IL-21**	3.0(2.1)	2.3	3.7(1.4)	3.9	3.0(1.8)	3.6	0.892
**IL-4**	0.6(0.4)	0.4	0.3(0.1)	0.4	0.5(1.2)	0.4	1.000
**IL-23**	29.2(15.7)	25.2	40.5(18.2)	37.8	32.6(20.0)	34.6	0.623
**IL-5**	3.2(1.1)	2.9	3.3(1.0)	2.9	3.7(0.8)	3.8	0.012
**IL-6**	5.6(2.7)	5.0	5.0(1.5)	5.4	4.7(1.2)	4.4	0.189
**IL-7**	6.9(5.3)	6.3	7.6(4.5)	7.9	8.6(5.1)	8.7	0.521
**IL-8**	25.8(5.3)	25.0	26.0(6.0)	25.6	30.5(5.8)	29.6	0.010
**TNF-alpha**	0.8(0.9)	0.4	0.6(0.4)	0.7	1.5(0.9)	1.5	0.015

^a^Kruskal-Wallis Test; SD—Standard deviation;; Me—median; AG—Asymptomatic Group; SG- Symptomatic Group; HG- HAM/TSP Group; CSF—Cerebrospinal Fluid.

The cytokines and chemokines that presented significant statistical difference between asymptomatic and HAM/TSP groups were: plasmatic ITAC (p = 0.003), CSF ITAC (p = 0.001) and IL-8 (p = 0.010). Symptomatic and HAM/TSP groups showed statistical difference only CSF ITAC (p = 0.003). Asymptomatic and symptomatic groups did not present significant statistical difference in relation to the cytokines and chemokines analyzed. Only plasma ITAC correlated with ITAC in CSF (N = 45; r_s_ = 0.56, p < 0.001). Plasmatic (N 47) and CSF (N 46) ITAC correlated with disease time (r_s_ 0.43; p = 0.003 and r_s_ 0.38; p = 0.009), ambulation index (r_s_ 0.44; p = 0.001 and r_s_ 0.46; p = 0.001), respectively, but not correlated with thoracic SUV.

Plasmatic ITAC (N = 44; U = 132.0, p = 0.034, r = −0.32; M_e_: 10.52 and 8.85), IL-5 (N = 44; U = 305.5, p = 0.029, r = 0.33; M_e_: 0.37 and 0.51) and CSF ITAC (N = 43; U = 930.0, p = 0.006, r = 0.41; M_e_: 71.51 and 45.87) presented significant statistical difference in relation to high or low blood proviral load, respectively. However, when only the asymptomatic group was analyzed, there was no statistically significant difference of the cytokines in relation to high or low blood proviral load.

### Variables correlated with EDSS

**Fig 4:**
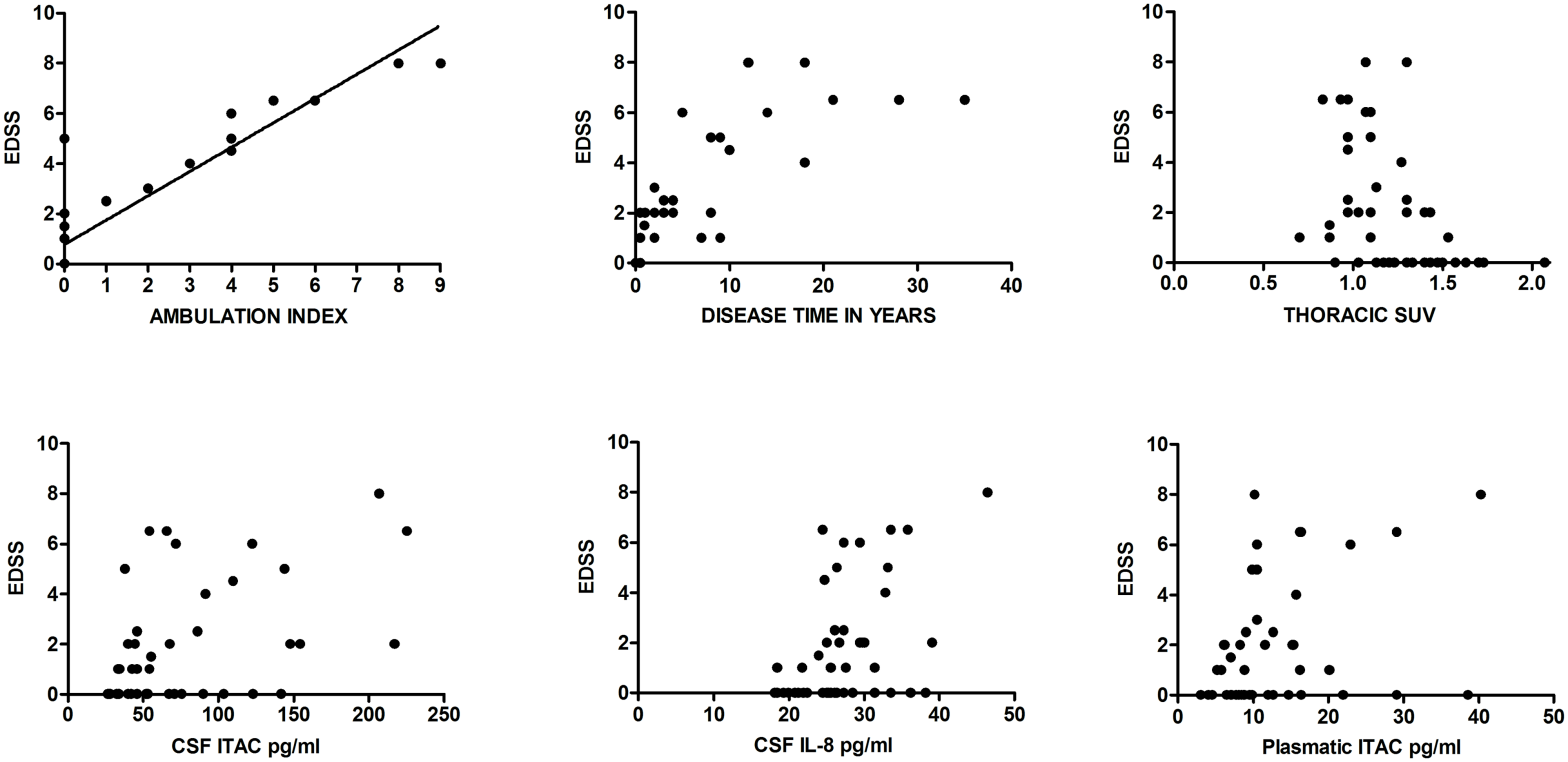
The graphs represent the clinical correlation measured by the EDSS score with other variables of interest.

EDSS correlated strongly with: disease time (N 48; r_s_ 0.95; p < 0.001); ambulation index (N 48; r_s_ 0.81; p < 0.001); thoracic SUV (N 48; r_s_ 0.50; p < 0.001); CSF ITAC (N 46; r_s_ 0.45; p 0.002) and CSF IL-8 (N 46; r_s_ 0.42; p 0.003) (Fig 4). A moderate EDSS correlation occurred with: CSF proviral load (N 41; r_s_ 0.40; p 0.011); plasma ITAC (N 47; r_s_ 0.37; p 0.012) CSF IL-5 (N 46; r_s_ 0.35; p 0.019); CSF IFN-gamma (N 46; r_s_ 0.34; p 0.021) and plasma IL- 17 A (N 47; r_s_ -0.35; p 0.016).

### Analysis of risk factors for the development of HAM/TSP

In this analysis, only the asymptomatic and HAM/TSP groups were considered, since the subjects of the symptomatic group were considered clinically undetermined. Univariate analysis indicated that female gender, thoracic spinal cord SUV, blood proviral load, plasmatic IL-6, CSF ITAC, IFN-gamma and IL-8 were predictors to HAM/TSP, when taken as independent risk factors. Only thoracic spinal cord SUV and CSF ITAC fit in the multivariate model, with a correct classification between asymptomatic and HAM/TSP individuals of 81.3%, as shown in Table 3.

**Table 3:** Risk factors predictive of HAM/TSP in univariate and multivariate regression.

VARIABLES	UNIVARIATE				MULTIVARIATE			
	B	OR	95% CI	*P*	B	OR	95% CI	*P*
Thoracic SC SUV	-6.904	0.001	0.001–0.118	0.011	-8.536	0.001	0.001–0.141	0.011
CSF ITAC	0.026	1.027	1.006–1.047	0.024	0.032	1.033	1.004–1.062	0.006
Female gender	1.754	5.778	1.258–26.53	0.024				0.124
Plasma IL-6	1.514	4.545	1.005–20.55	0.049				0.141
CSF INF-gamma	0.608	1.836	1.041–3.238	0.036				0.323
CSF IL-8	0.165	1.179	1.014–1.372	0.033				0.421
Blood proviral load	0.004	1.004	1.000–1.008	0.050				0.312

Multivariate conditional backward stepwise (Wald) model with imput *p* = 0.100, constant *p* = 0.048. Adjusted R^2^ of the multivariate model: 0.654. Hosmer-Lemeshow test, *p* = 0.297.

## Discussion

In this study we sought to better understand the HTLV-1 clinical manifestations and its correlations with imaging and immune features. We enrolled and classified infected individuals according to their neurological status in asymptomatic, symptomatic and HAM/TSP groups. These groups were statistically different regarding neurological symptoms and signs, EDSS score and gender, but showed no difference regarding age and distribution of systemic diseases. Female gender was more frequent in the symptomatic (4.5:1) and HAM/TSP groups (4.3:1). This higher proportion of female gender in the symptomatic and HAM/TSP groups is explained by the higher incidence and prevalence of HAM/TSP in females, as observed in other epidemiological studies [21]. For this reason, statistical analyzes of the variables described in this paper were also analyzed in relation to gender, with the purpose of evaluating a possible gender influence, which was not found.

First, we discussed two questions. Are there HTLV-1 infected individuals without CNS impairment? Is HTLV-1 related CNS impairment restricted to the thoracic spinal cord? Analyzing this same population (Schütze et al., 2017), we divided the sample according to the EDSS score (0; 1–2; 2.5–10) and processed each PET brain image using the statistical parametric mapping toolbox. Gray and white brain matter density image were generated for each subject. Analysis was able to classify with the accuracy of 85.7% (p = 0.001), 75% (p = 0.024) and 85% (p = 0.003) groups with EDSS score 0 (asymptomatic), 1–2.5 (intermediate) and > 2.5 (HAM/TSP), respectively, in relation to the control group. Diffuse cerebral hypometabolism was observed in HTLV-1 infected groups, including those with zero EDSS score compared to controls [22]. Other studies have shown that additional neurological manifestations are present in HTLV-1 infection other than HAM/TSP and that other segments of the central nervous system beyond the thoracic spinal cord, such as: brain and cervical spinal cord are altered even in infected HTLV-1 considered asymptomatic. [5–6, 13–15, 22]. The results of 18-F-FDG PET/CT associated with clinical data lead us to conclude that in most HTLV-1 infected individuals there is involvement of the evaluated structures of CNS, despite lack of clinical symptoms.

The 18F-FDG PET/CT study shown hypometabolism in the cervical and thoracic segments of the spinal cord in all the three groups of HTLV-1 infected individuals including the group considered asymptomatic compared to controls. The 18-F FDG PET/CT was able to show greater involvement of the thoracic spinal cord, with excellent accuracy in the discrimination between HTLV-1 infected individuals (81.6%) and HAM/TSP patients (94.4%) from control group, as well as between HTLV-1 infected asymptomatic and HAM/TSP groups (85.7%).

Currently, the major pathophysiological mechanism considered to explain CNS involvement in HAM/TSP is an immuno-mediated chronic inflammatory process in response to HTLV-1 infection, which damages nearby CNS components [7]. We expected that 18F-FDG PET/CT showed in the initial phase of neurological impairment, prior to the neurodegenerative process, a metabolic increase due to inflammation. However, HTLV-1 infected individuals showed hypometabolism in 18F-FDG PET/CT in comparison to the control group, including HTLV-1 infected individuals with none neurological symptoms. CNS 18F-FDG PET/CT hypometabolism was observed in brain small-vessel and neurodegenerative diseases such as in preclinical stages of Alzheimer’s disease. This hypometabolism finding is compatible with GLUT-1 expression reduction, a metabolic disorder and/or a vascular-neuronal dysfunction coursing with consequent breakdown of the blood-brain barrier and degeneration [23–24]. This hint lead us to rethink the process to explain the lesions of CNS in HTLV-1 infection.

The 18F-FDG behaves like glucose in the vascular and interstitial compartments and is transported to the intracellular compartment by passive transport, using glucose transporter proteins (GLUT), especially GLUT-1, present in most tissues and in the CNS. In the cell, 18-F FDG is phosphorylated and trapped in the intracellular compartment allowing the measurement of tissue metabolism [25]. PET/CT hypometabolism may result from low tissue metabolism, hypoperfusion due to low tissue blood supply, GLUT-1 reduction or blockage of glucose entry to the intracellular environment [23]. Why asymptomatic and symptomatic infected HTLV-1 have spinal cord hypometabolism despite of not presenting neurological impairment on physical examination? Why the symptomatic group have hypometabolism in a similar degree to the HAM/TSP group? Multiple mechanisms may be responsible for hypometabolism and we do not know for sure what causes this process in the pre-clinical and advanced stages of HAM/TSP. As already described above, in other pathologies demonstrating 18F-FDG PET/CT hypometabolism, this finding is apparently associated with microvascular changes, loss blood-brain barrier homeostasis and its consequences [23–24]. In addition, pathological and 18F-FDG PET studies have shown that less perfused areas of the central nervous system (watershed) are more vulnerable to HTLV-1 infection [26–27] and that GLUT-1 is important in the cell-entry mechanisms of HTLV-1 associated the vascular endothelial growth factor (VEGF), Neuropilin-1 (NRP-1) and heparan sulfate proteoglycans (HSPG) [28]. These areas of the central nervous system with lower perfusion may have higher VEGF expression, with higher HSPG and NRP-1 exposure on the endothelial cell surface, allowing HTLV-1 cell-entry [29]. These changes would promote imbalance in angiogenic homeostasis and the rupture of the local blood-brain barrier [30]. The breakdown of the blood-brain barrier allows the passage of infected and uninfected lymphocytes into the central nervous system tissue with subsequent local immune response, inflammation, demyelination and axonal degeneration [30]. However, this is a speculative hypothesis and we know that further studies are still necessary to have a clue to explain the mechanisms for the observed CNS and spinal hypometabolism.

High proviral load is a known risk factor for HAM/TSP development [31–32]. The individuals with HAM/TSP seem to have a higher blood and CSF proviral load, but there are not a cut-off point and reproducibility between the studies [33]. In this study, blood and CSF proviral load were not predictors of HAM/TSP in multivariate regression. There was a strong unidirectional positive correlation between the blood and CSF proviral load, with an average increase of CSF proviral load of nine times and median of 3.5 times over the blood proviral load, but there is none difference among the three HTLV-1 infected groups and, no correlation between blood and CSF proviral load and thoracic spinal cord SUV. In the other hand, symptomatic group had the lower blood and CSF proviral load and their thoracic spinal cord SUV was similar to that observed in HAM/TSP group (p = 1.00). This finding suggests a possible incipient neurological involvement in individual with low proviral load. This evidence reinforces the lack of association between proviral load and spinal cord hypomethabolism.

About the immune profile, there is no specific immunological marker for diseases associated with HTLV-1 infection [7, 34]. The cytokines and chemokines evaluated did not differ between asymptomatics and symptomatics, but both groups showed differences compared with HAM/TSP. HAM/TSP have higher rate of plasmatic ITAC, CSF ITAC and IL-8.

ITAC is induced by INF-gamma and it plays an important role in leucocyte trafficking, principally acting on activated CD4 + Th1 cells, CD8 + T cells and NK cells [35]. IL-8 has chemotactic properties on neutrophils and other granulocytic cells, and is also related to angiogenesis [36]. In this study, IL-17A and IL-21, Th17 pathway activity biomarkers, had lower levels in the HAM/TSP compared to asymptomatic and symptomatic. Th17 pathway is involved in distinct pathophysiological mechanisms of inflammatory, infectious, autoimmune and neoplastic diseases [37, 38]. We then observed that HAM/TSP group showed CSF inflammatory Th1 profile, with apparent plasmatic Th17 pathway suppression compared to asymptomatic group. Rosa et al. 2018 published an analysis of the cytokine and chemokine profile in the plasma and CSF of this same population classified into two EDSS-based groups: EDSS score 0 and > 0. Data analysis also demonstrated a Th1 pro-inflammatory profile in patients’ CSF with EDSS score > 0 [39].

In the regression analysis, several independent predictors did not adhere to the multivariate model. It is possible that this was due to the small sample number and the multiple correlations observed between cytokines, chemokines and proviral load. Thoracic spinal cord SUV and CSF ITAC that adhered to the multivariate model were jointly good predictors of HAM/TSP, correctly classifying 81.3% individuals into asymptomatic and HAM/TSP groups. The results of multivariate logistic regression predicted that for a decrease of each unit of thoracic SUV, there was an increase of 99 times at risk of HAM/TSP and for each unit of increase of CSF ITAC, the risk of HAM/TSP increases 0.33 times.

We are aware of limitations of our study, such as the small sample size, and the not HTLV-1 serological testing in PET/CT control group. Regarding this last point, we considered a very low risk of HTLV-1 infection in the control group given that the estimated prevalence of this infection in the general Brazilian population is around 1% [4]. However, possible HTLV-1 infected individuals in this group would reduce the magnitude of the effect observed in 18F-FDG PET/CT.

In conclusion, HTLV-1 infection involves the CNS of most infected individuals even in absence of clinical symptoms. The thoracic spinal cord is definitely affected by HTLV-1, but other CNS segments are also compromised. 18F-FDG PET/CT seems a promising diagnostic tool to evaluate HTLV-1 CNS commitment, especially when associated to high CSF-ITAC levels might explain clinical outcome in different individuals. Our findings suggest that HTLV-1 infection may promote CNS vascular-neuronal dysfunction, propitiating breakdown of CNS protection barriers, lymphocytic infiltration with pro-inflammatory response that contributes concomitantly to neuronal damage. CNS segments with lower blood flow are more susceptible to vascular-neuronal dysfunction, which may be related to HTLV-1 cell-entry mechanisms. Genetic or acquired pathologies related to microcirculatory angiogenesis may be a possible factor to explain the development of HAM/TSP in specific and familial population groups. Further investigation is necessary to assess possible microcirculatory impairment and immune mediated mechanism in HAM/TSP pathophysiology. Our findings showed new venues for studies about potential metabolic and microvascular involvement in the neurological impairment of HTLV-1 infected individuals.

## Material and methods

### Ethics statement

All the procedures performed in the study, involving human participants, were approved by the Research Ethics Committee of the two participating institutions: Hemominas Foundation and Federal University of Minas Gerais, according to the 1964 Helsinki Declaration and its subsequent amendments.

Informed written consent was obtained from all participants included in the study.

### Study design

This is a cross-sectional study of a cohort of HTLV-1 infected individuals and a nested case-control study between HTLV-1 infected and not infected controls in relation to spinal cord analysis by 18-F FDG PET/CT.

### Population

The sample was non-probabilistic and composed of 50 individuals participating in the GIPH cohort, Belo Horizonte, Minas Gerais, Brazil. The GIPH cohort has a record of 1,200 participants among former blood donors diagnosed with HTLV-1 and 2 infection, their sexual partners, relatives, patients referred, and controls. It is an open cohort started in 1997. The sample for this study was a group of 300 HTLV-1 infected cohort participants who underwent neurological evaluation performed between 2007 and 2014. Participants were admitted in the period from January 2013 to October 2015 and data was collected and analyzed until December 2016.

Two of the 50 subjects invited for this study were excluded. One due to HIV co-infection and the second although showed positive ELISA test for HTLV-1, confirmatory WB was inconclusive and qualitative PCR in blood was negative in two tests. The 48 HTLV-1 infected individuals who remained in the study were subdivided in three groups, according to neurological impairment, observed in the first appointment. Complementary tests were performed up to 60 days after this appointment. The group referred to as asymptomatic (AG) was formed by participants who did not present any symptom, or just few or common symptoms observed in the general population, insufficient to characterize neurological impairment. All individuals classified asymptomatic had a normal neurological physical examination (Fig 1). The symptomatic group (SG) consisted of individuals with symptoms (Fig 1) and normal neurological physical examination. The HAM/TSP group (HG) had individuals that fulfilled the established diagnostic criteria for HAM/TSP (Fig 1) [8–9]. The control group was paired for sex and age with the case-group. The control group was composed by 10 healthy individuals and 20 individuals with prostate and colon cancer without CNS involvement, that were submitted to 18-F FDG-PET/CT imaging for other reasons. All individuals enrolled gave written informed consent and allowed their PET/CT image to be used in scientific research.

The sample (**AG** and **HG**) evaluated in this study is representative when considering the magnitude of the effect between the symptoms and signs observed in asymptomatic (<10%) and HAM/TSP group (> 50%), for α = 0.05 (bilateral) and β = 0.20. Sixteen individuals per group were predicted. The same cannot be attributed to the symptomatic group.

The inclusion criteria were: HTLV-1 infection confirmed by Western blotting (WB HTLV 2.4, Genelabs Diagnostics, Singapore) or real-time PCR; four or more years of formal education, minimum of 18 and maximum of 70 years old. Co-infection evidenced by positive serological tests for human immunodeficiency virus, hepatitis C and B virus, Chagas disease, syphilis, neurological impairment or disease due to another etiology were exclusion criteria.

### Data collection and procedures

**Fig 5:**
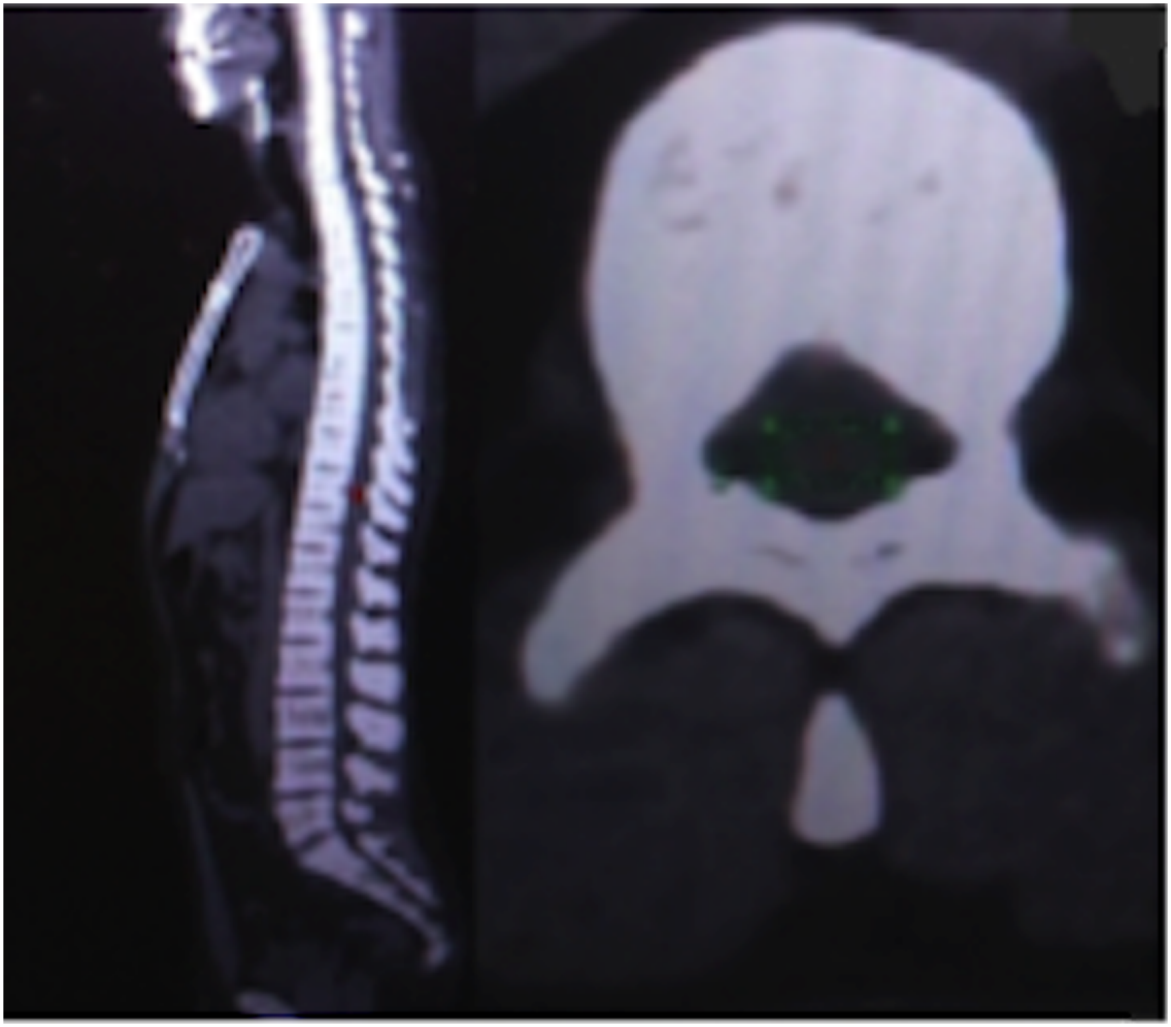
Sagital (left) and axial (right) PET/TC image. Area of measurement of the SUV, plane axial (green).

The study was performed in four steps: **Clinical**—Individuals of the GIPH cohort, eligible for the project, were called for an individual appointment. In this first contact they were informed about project objectives and tests. Those interested in participating signed the Term of Consent and then underwent a neurological appointment, neuropsychological assessment and blood collection to perform serology of possible co-infections. The clinical and neurological data collected in the appointment were systematized and recorded into a database built in the Epidata 3.1 software. **Complementary tests**–Blood and CSF proviral load, cytokines, chemokines and 18F-FDG PET/CT of the whole body were performed at the Molecular Imaging Center (CTMM/UFMG). The participants were instructed to fast for at least 6 hours. First, capillary glycemia was measured, venous access was obtained and used to collect blood for proviral load, cytokines and chemokines assays. Venous access was maintained with 0.9% saline until the end of the procedure. Subsequently, participants received a 18F-FDG radiotracer bolus, 5.18 MBq/Kg, by intravenous administration. Participants were kept resting for 50 minutes in a room with dimmed light and low stimulation, prior to acquisition of PET/CT images in a time interval of approximately 10 minutes, and reconstructed in a 192 x 192 x 47 matrix using the OSEM (Ordered Subsets Expectation Maximization) algorithm, with 2 iterations and 20 subsets. Attenuation correction was performed using computed tomography (CT) images. A specific protocol was performed for targeted analysis of the central nervous system, especially the spinal cord. Standardized uptake value (SUV) of the thoracic and cervical spinal cord was measured. In the thoracic spinal cord, SUV was measured on T6, T8 and T10 levels, and at the cervical spinal cord on C5, C6 and C7 levels. The spinal cord levels were located using the CT image, having the sagittal plane to define the levels and axial plane to locate the medulla in the central canal of the spinal column. The SUV was measured in an oval area with 12 mm and 6 mm diameter, lateral-lateral and anterior-posterior, respectively (Fig 5). The PET software provided the average SUV measured in the oval area of the particular spinal cord segments. SUV average of the three cervical and three thoracic spinal cord segments were used in the statistical analysis.

After the acquisition of PET/CT images, participants underwent a lumbar puncture with local anesthesia for collection of 8 to 10 ml of CSF. Immunobead assay Milliplex MAP human high sensitivity T cell assay (EMD Millipore) was used to quantify the expression levels of 19 cytokines in plasma and CSF. The HSTCMAG-28SK kit was designed to detect the following analytes: ITAC, GM-CSF, Fractalkine, IFN-gamma, TNF-α, MIP-3α, IL-1b, IL-12, IL-13, IL-17A, IL-2, IL-4, IL-5, IL-6, IL-7, IL-8, IL-10, IL-21 and IL-23. Plates were analyzed using a Luminex100/200 system. Median fluorescence intensity (MFI) was converted to concentration (in pg/ml) using an equation derived from the standard range of each analyte using Milliplex Analyst Software. Each sample was measured in duplicate. Cytokines in the blood and CSF results were not obtained in some HAM/TSP (blood 1 and CSF 2) due to technical difficulties in collecting the material or in the processing of the test. Proviral load in blood and cerebrospinal fluid material were immediately centrifuged at 3,000x g for 5 minutes to obtain the buffy coat layer and the cell pellet, respectively, used to obtain DNA. The DNA was extracted using the QIAamp DNA Blood Kit (Qiagen GmbH, Hilden, Germany), following manufacturer’s instructions. The quantification of proviral load was performed by real-time PCR using SYBR Green. Real-time PCR was performed on the ABI Prism 7300 Sequence Detector System (Applied Biosystems). The proviral charge value was calculated as [(average number of copies pol/average number of albumin copies/2)] x 10,000 and expressed as the number of proviral copies/10,000 cells. The proviral load in blood and CSF were not obtained in some participants due to technical difficulties during collection of the material or the processing of the test: asymptomatic (blood 1 and CSF 3) and HAM/TSP (blood 2 and CSF 4).

### Collection of data and procedures

Demographic, clinical and laboratory data were collected and analyzed using the SPSS 22.0. The descriptive analysis of the groups included the distribution of frequency and percentage of categorical variables, measures of central tendency according to the best suitability for quantitative variables. The comparative analysis between groups included X^2^ test or Fisher test for categorical variables, t test or ANOVA for quantitative variables with normal distribution, Kruskal-Wallis and Mann-Whitney test for non-parametric data. Pearson or Spearman correlation coefficients were used according to the parametric or non-parametric distribution of the data, respectively. The ROC curve was used to calculate the accuracy, sensitivity, specificity and cutoff of some variables. In order to define which variables would form the best predictor model for HAM/TSP, univariate and multivariate regression were performed. Statistical significance was considered when p ≤ 0.05, except for multivariate entries where p = 0.10 and Bonferroni correction where p = 0.0167 were considered. The graphics were edited using the software: GraphPad Prism 7.0d and Microsoft Exel 2008 for Mac, version 12.3.1.

## Supporting information

S1 Checklist STROBE checklist. (DOCX)
