# Supplementary material for: Spinal cord hypometabolism associated with infection by human T-cell lymphotropic virus type 1(HTLV-1)
Source: PLoS Negl Trop Dis. 2018 Aug 27;12(8):e0006720. doi: 10.1371/journal.pntd.0006720 (PMC6128630; doi:10.1371/journal.pntd.0006720)
Supplement: S1 Checklist — (DOCX) [file pntd.0006720.s001.docx]

STROBE STATEMENT SUPPORTING INFORMATION LEGENDS

| ITEM | SECTION | SUBSECTION | PARAGRAPH |
| --- | --- | --- | --- |
| S1a | Abstract | Methodology | 1 |
| S1b | Abstract | Methodology and pricipal findings | 1 |
| S2 | Introduction |  | 2, 3, 4 |
| S3 | Introduction |  | 4 |
| S4 | Methods | Study design | 1 |
| S5 | Methods | Population | 1 |
| S6 | Methods | Population | 1, 2, 4 |
| S7 | Methods | Population | 2 |
| S8 | Methods | Population | 2 |
| S9 | Discussion |  | 1 |
| S10 | Methods | Population | 3 |
| S11 | Methods | Data collection and procedures | 1 |
| S12 | Methods | Data collection and procedures | 1 |
| S13 | Results | Demographic and clinical data | 1 |
| S14 | Results | Demographic and clinical data | 1 |
| S15 | Results | Spinal cord standardized uptake value (SUV) | 1,2,3 |
| S16 | Results | Spinal cord standardized uptake value (SUV) | 1,2,3 |
| S17 | Results | Blood and CSF Proviral Load  Cytokine and chemokine biomarker  Variables correlated with EDSS  Analysis of risk factors for the development of HAM/TSP | 1  1,2,3  1  1 |
| S18 | Discussion |  | 2,3,9,11 |
| S19 | Discussion |  | 10 |
| S20 | Discussion |  | 5 |
| S21 | Discussion |  | 11 |
| S22 | Discussion |  | 11 |
